# Ultrahigh-speed graphene-based optical coherent receiver

**DOI:** 10.1038/s41467-021-25374-0

**Published:** 2021-08-20

**Authors:** Yilun Wang, Xiang Li, Zhibin Jiang, Lei Tong, Wentao Deng, Xiaoyan Gao, Xinyu Huang, Hailong Zhou, Yu Yu, Lei Ye, Xi Xiao, Xinliang Zhang

**Affiliations:** 1 Wuhan National Laboratory for Optoelectronics and School of Optical and Electronic Information, Huazhong University of Science and Technology, Wuhan, China; 2 Electrical Engineering Division, School of Engineering, University of Cambridge, Cambridge, UK; 3 State Key Laboratory of Optical Communication Technologies and Networks, China Information and Communication Technologies Group Corporation (CICT), Wuhan, China; 4 National Information Optoelectronics Innovation Center, Wuhan, China

**Keywords:** Nanophotonics and plasmonics, Fibre optics and optical communications, Integrated optics, Optical properties and devices

## Abstract

Graphene-based photodetectors have attracted significant attention for high-speed optical communication due to their large bandwidth, compact footprint, and compatibility with silicon-based photonics platform. Large-bandwidth silicon-based optical coherent receivers are crucial elements for large-capacity optical communication networks with advanced modulation formats. Here, we propose and experimentally demonstrate an integrated optical coherent receiver based on a 90-degree optical hybrid and graphene-on-plasmonic slot waveguide photodetectors, featuring a compact footprint and a large bandwidth far exceeding 67 GHz. Combined with the balanced detection, 90 Gbit/s binary phase-shift keying signal is received with a promoted signal-to-noise ratio. Moreover, receptions of 200 Gbit/s quadrature phase-shift keying and 240 Gbit/s 16 quadrature amplitude modulation signals on a single-polarization carrier are realized with a low additional power consumption below 14 fJ/bit. This graphene-based optical coherent receiver will promise potential applications in 400-Gigabit Ethernet and 800-Gigabit Ethernet technology, paving another route for future high-speed coherent optical communication networks.

## Abstract (web summary)

Graphene-based photodetectors have many advantages for applications. Here, the authors demonstrate a high-speed optical coherent receiver for optical communications based on graphene-on-plasmonic slot waveguide photodetectors.

## Introduction

Graphene-based optoelectronic devices^1,2^, such as high-speed photodetectors^3^ (PDs) and modulators^4^, take advantage of graphene’s high carrier mobility^5^, ultra-broadband spectral response^6,7^, and integration capability with on-chip photonics platforms^8^, showing blooming application prospects in the information age. Integrated graphene-based PDs with high-speed (~500 GHz^3^) performance, compact footprint, and high responsivity are significant for microwave photonics^9^, terahertz communication^10^, and optical communication^11^. In the field of waveguide-integrated graphene-based PDs, different mechanisms (photovoltaic^12,13^, photothermoelectric^14,15^, photoconductive^16^, and photobolometric^17^), broadband detection^18,19^, responsivity of ~0.5–0.7 A/W^20,21^ and high-speed up to 110 GHz^20,22^ were demonstrated. In particular, the integrated graphene-based PDs enabled by the plasmonic structures^16,20–25^ on silicon platform have attracted a great deal of attention recently, attributed to the greatly enhanced light–graphene interaction, and high-speed characteristics of plasmonic structures.

Currently, plasmonic waveguide-integrated graphene-based PDs with a compact size of a few microns^20,21^ show a high responsivity up to 0.7 A/W^21^, a large bandwidth of 110 GHz^20,22^, and a high receiving capacity of 100–120 Gbit/s^20,26,27^. However, graphene-based PDs are still difficult to be applied in 200-Gbit/s, 400-Gbit/s, or even higher 800-Gbit/s optical interconnections^28^, while direct detection mode is exploited. To meet the growing capacity demands of global data traffic, coherent optical communication^29^ adopting advanced modulation formats and digital signal processing (DSP) can increase the spectral efficiency and the data transmission capacity^30–33^. At the receiving end of the coherent optical communication system, the optical coherent receiver (OCR)^34–37^ that combines a 90-degree optical hybrid and four PDs (single polarization), can decode the information carried on the amplitude and phase of the light, achieving the multiple increases of the receiving capacity. Meanwhile, by utilizing the gain provided by the local oscillator (LO) and balanced detection, the OCR can decrease noises influences and improve detection sensitivity. Based on plasmonic waveguide-integrated graphene-based PDs and coherent detection, the optical receiver will have a chance to realize a large bandwidth and a high-quality large-capacity data reception. However, at present, graphene-based OCRs have not yet been experimentally demonstrated.

Here, we present a single-polarization graphene-based OCR enabled by plasmonic slot waveguides (PSWs) with compact active detection sections of 4 × 15 μm × 100 nm (four graphene-on-PSW PDs^26^ with an active detection area of 15 μm × 100 nm each (see Supplementary Note 1 and Supplementary Fig. 3)) and a simple fabrication process. Each graphene-on-PSW PD exhibits a responsivity of >0.1 A/W and a large bandwidth far exceeding 67 GHz (limited by the test equipment) under a low working voltage of −0.3 V. Moreover, the advanced modulation formats, including binary phase-shift keying (BPSK) up to 90 Gbit/s, quadrature phase-shift keying (QPSK) up to 200 Gbit/s (100 Gbaud), and 16 quadrature amplitude modulation (QAM) up to 240 Gbit/s (60 Gbaud), are successfully detected by the proposed graphene-based PDs with power consumption below 14 fJ/bit. This proposed OCR implemented on a different-material mechanism takes the advantage of the combination of graphene optoelectronics, plasmonic sub-wavelength light confinement, and silicon photonics, all of which allow for efficient and high-speed detection on the μm-scale footprint.

## Results

### Device design and fabrication

**Fig. 1:**
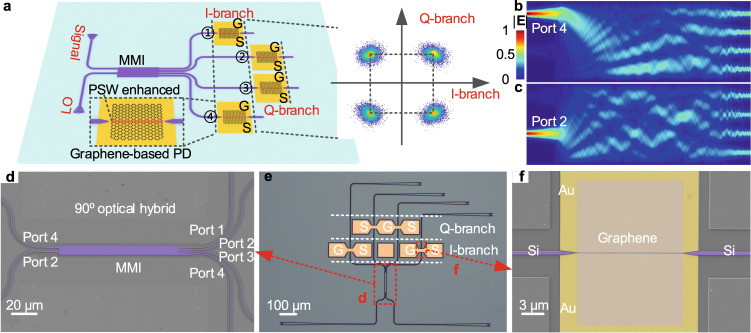
The OCR with graphene on PSW. **a** Schematic diagram of the proposed graphene-based OCR. GS electrodes: ground-signal electrodes. **b**, **c** The mode profile of the MMI coupler under the light input from port 4 (**b**) and port 2 (**c**), respectively. **d** False-color SEM image of the MMI coupler (90-degree optical hybrid). **e** Optical microscopy image of the proposed OCR. The image was taken before transferring graphene to the device. **f** False-color SEM image of a graphene-on-PSW PD.

Figure 1a illustrates the working principle of the proposed graphene-based OCR, which is composed of a 90-degree optical hybrid and four graphene-on-PSW PDs. The 90-degree optical hybrid here utilizes a common 4 × 4 multimode interference (MMI) coupler, which works in the self-imaging principle^38^ to control the phase of the output-port light (see Supplementary Note 1, Supplementary Figs. 1,2, and Supplementary Table 1). The optical simulation of the MMI coupler is implemented through the finite-difference time-domain method. Figure 1b, c show the mode profiles of the MMI coupler under the light input from port 4 and port 2, respectively. After the signal light and the LO light input from port 4 and port 2 on the left side of the optical hybrid, respectively, the radio frequency signals with high-order modulation that encoded on the signal light can be balance-detected by the PD1 and PD4 (PD2 and PD3) as in-phase (quadrature) (I/Q) components because of a 180-degree phase difference. After the DSP, the original advanced modulation format can be integrally acquired with high quality. Besides, PD1 (PD4) or PD2 (PD3) can respectively implement the detection of one I-branch or Q-branch, thus utilizing the combination of two PDs from the I- and Q-branches can achieve the coherent detection of the original information without balanced detection. It is worth noting that our graphene-based PDs works at only a fixed bias voltage, resulting in a more straightforward operation with no more than four bias voltages to realize the detection of advanced modulation formats.

The devices are fabricated in-house (see Methods) to demonstrate the scheme. The optical microscope image of the proposed OCR is shown in Fig. 1e. In the actual device fabrication and measurement, two input grating couplers are used to input the signal light and the LO light, respectively. The output grating couplers are utilized to test the transmission loss of the device and monitor the coupling state of the input light. To avoid the signal delay, time sequence mismatch and additional loss imbalance between the four channels, the length of the silicon waveguide from the MMI output ports to the input ports of PDs is consistent to ensure the four paths have the same optical path length. Figure 1d, f show the false-color scanning electron microscopy (SEM) images of a 90-degree optical hybrid with a size of 114.5 μm × 8 μm and a graphene-on-PSW PD with 15-μm-long graphene covering the PSW, respectively. The transverse-electric light in the silicon waveguide is converted into the plasmonic mode in the PSW by the silicon–plasmonic tapered coupler^39,40^. This plasmonic mode can enhance the light–graphene interaction to improve the responsivity of the graphene-based PD. The length of the tapered coupler is 2.06 μm, the metallic part of the PSW is 5 nm/90 nm Ni/Au, and the slot width and length are 100 nm and 15 μm, respectively. The contact electrode occupies an area of 100 μm × 100 μm to facilitate the test, but it is not necessary when integrated with electronics. The PSW with this compact footprint can provide ultra-small resistance-capacitance parameters and ultra-short photo-generated carrier drift channel, resulting in a large bandwidth of graphene-on-PSW PDs. The transmission losses of single silicon–plasmonic tapered coupler and coupling grating were measured to be 3.4 and 3.2 dB at 1550 nm, respectively (See Supplementary Fig. 7). By optimizing the structure of the tapered coupler^32^ and adopting the edge-coupling method^41^, the additional loss from the optical fiber to the detection area can be further reduced.

### Device characteristics

**Fig. 2:**
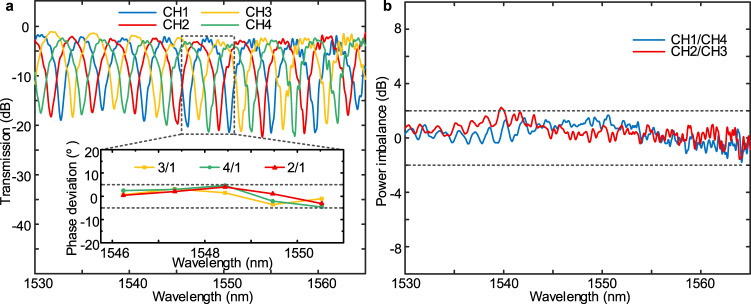
The 90-degree optical hybrid performance. **a** Measured transmission of the four output ports in the hybrid. CH stands for the output channel, and the number corresponds to the output port number. Inset: Measured phase deviation of the hybrid. **b** Measured power imbalance of the hybrid. The blue line (red line) represents the power imbalance between the output channels 1 and 4 (channels 2 and 3).

The detailed performances of the fabricated optical hybrid and PDs in the graphene-based OCR are measured as follows. Cascaded with a 1 × 2 MMI coupler and an optical delay line (see Supplementary Fig. 5f), the phase information of the output light in the referenced optical hybrid can be transferred into the intensity, as shown in Fig. 2a. The phase deviations of the output ports in the optical hybrid are further calculated as shown in the inset of Fig. 2a. The phase deviations between the output port 2/3/4 and the output port 1 are less than 5 degrees in the wavelength range of 1546–1551 nm. Meanwhile, the power imbalances among the output ports can be obtained through testing the transmissions of a reference optical hybrid (see Supplementary Fig. 5g). As shown in Fig. 2b, the power imbalances between the output port 1 and port 4 (or the output port 2 and port 3) of the optical hybrid are less than 2 dB in the C-band. This optical hybrid composed of a 4 × 4 MMI coupler does not require an additional phase control unit, resulting in a simple fabrication process and a convenient experimental setup. Moreover, the measured phase deviations and power imbalances of our optical hybrid can be easily compensated by the DSP^42^ for a high-quality coherent detection in a system experiment.

**Fig. 3:**
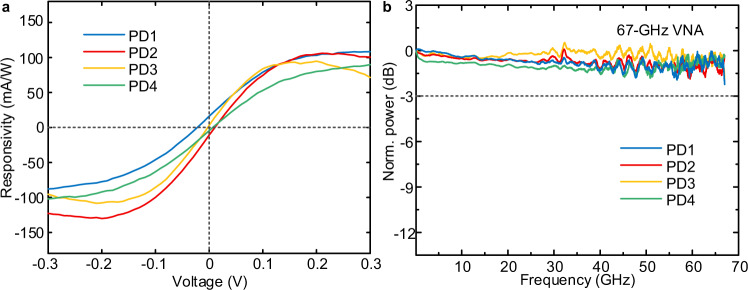
The performances of graphene-on-PSW PDs. **a** The responsivity as a function of the bias voltage for the four PDs at 1550 nm. **b** Frequency responses of the four PDs. VNA: vector network analyzer.

The static photo-responses of the four graphene-on-PSW PDs of the OCR are measured with the aid of a femto-ampere precision source (Keysight B2902A). Under the 1-dBm input light power entering the detection area, the relationships between the responsivities of the four PDs and the bias voltages are shown in Fig. 3a. The responsivity of either PD is close to 0.1 A/W at the −0.3 V-bias voltage. The lines in Supplementary Fig. 9 show that the photocurrents increase with the light input, indicating the dominated photoconductive effect^20,43^ in graphene-based PD without additional gate voltage control. However, some differences may exist in the contact barriers between graphene and both side electrodes for four PDs, resulting in nonzero and slightly different responsivity of four PDs at zero bias voltage. Still, they do not interfere with the performance of the coherent detection.

In the subsequent small-signal bandwidth measurement (S21 parameter), the working voltage of PDs is set at –0.3 V to improve the signal-to-noise ratios of the frequency responses. For the OCR, the measured 3-dB bandwidths of the four PDs shown in Fig. 3b are all greater than 67 GHz, which exceeds the measurable range of our vector network analyzer (Keysight N5227A). The bandwidth effects of the optical modulator, radio frequency cables, and high-speed probe are removed by utilizing calibration kits. The proposed PDs show a low power consumption below 14 fJ/bit contributed by a low working voltage (see Methods) and a large bandwidth performance beyond 67 GHz, which are very suitable for high-speed data reception of 100 Gbaud. In addition, the four PDs are separately utilized to realize the reception of 72 Gbit/s non-return-to-zero signal with a bit error ratio (BER) of less than 4.6 × 10^−4^ (see Supplementary Fig. 8).

### Data reception

**Fig. 4:**
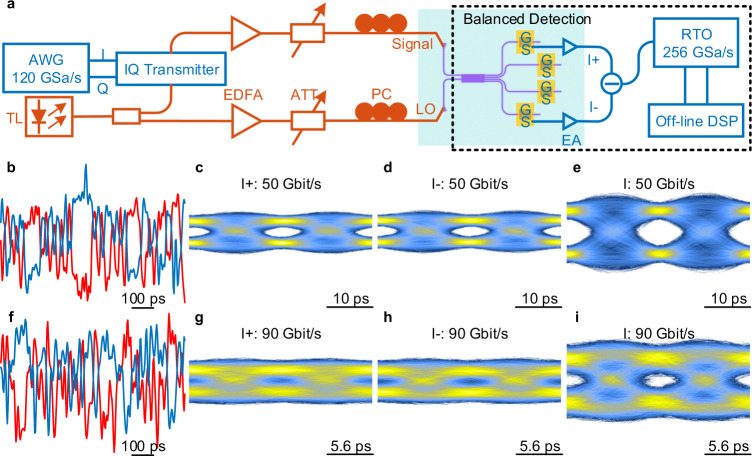
Balanced detection testing. **a** Measurement setup for the balanced detection. AWG: arbitrary waveform generator, TL: tunable laser, EDFA: erbium-doped fiber amplifier, PC: polarization controller, ATT: optical attenuator, EA: electric amplifier, RTO: real-time oscilloscope. **b**, **f** The real signal waveforms of 50 Gbit/s (**b**) and 90 Gbit/s (**f**) BPSK signals (blue line stands for I + branch and red line stands for I− branch). **c**–**e** Eye diagrams of I + branch (**c**), I− branch (**d**), and balanced detection (**e**) for 50 Gbit/s BPSK signal. **g**–**i** Eye diagrams of I + branch (**g**), I− branch (**h**), and balanced detection (**i**) for 90 Gbit/s BPSK signal.

For the high-speed data reception experiment (see Methods), we firstly implement BPSK signal receptions to verify the balanced detection of the proposed graphene-based OCR, with the experimental setup shown in Fig. 4a. 50 Gbit/s and 90 Gbit/s BPSK signals are successfully detected by PD1 and PD4, and the real signal waveforms and eye diagrams are demonstrated in Fig. 4b–i. As shown in Fig. 4b, f, the signal waveforms of 50 Gbit/s and 90 Gbit/s collected by the real-time oscilloscope show obvious 180-degree phase differences detected by two PDs. In Fig. 4c, d, g, h, the eye diagrams are detected by only one PD, and in Fig. 4e, i, the eye diagrams are detected by two PDs together through the balanced detection. The comparisons of these eye diagrams show that the signal-to-noise ratios of the received signals can be significantly improved by the balanced detection.

**Fig. 5:**
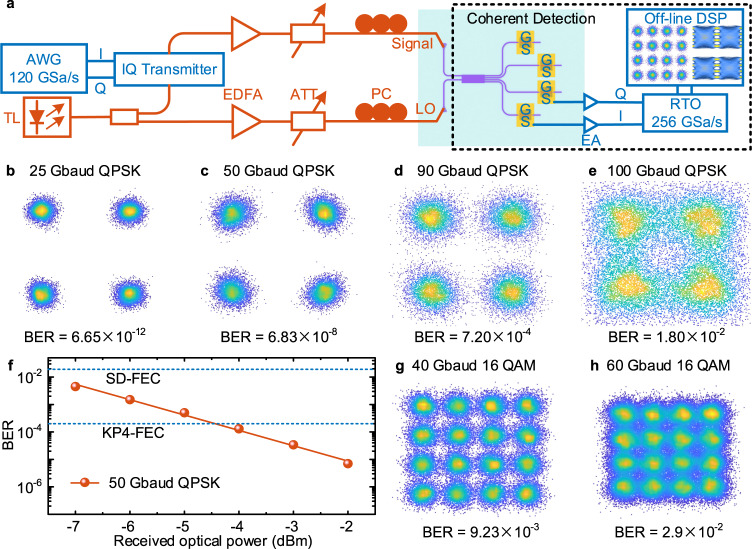
Experimental demonstration of coherent detection. **a** Measurement setup for the coherent detection. **b**–**e** Constellation diagrams of 25 Gbaud (**b**), 50 Gbaud (**c**), 90 Gbaud (**d**), and 100 Gbaud (**e**) QPSK signals. **f** Measured BERs versus the received optical power for 50 Gbaud QPSK signal. SD: soft-decision. **g**–**h** Constellation diagrams of 40 Gbaud (**g**) and 60 Gbaud (**h**) 16 QAM signals.

Then, we use the graphene-based OCR to realize single-polarization, advanced modulation formats reception up to 240 Gbit/s at 1550 nm. As the experimental setup shown in Fig. 5a, the PD3 and PD4 are utilized to detect the I- and Q-branches of the advanced modulation formats, owing to the limit of the measurement equipment. Although no balanced detection is performed, high-quality advanced modulation formats are still detected by adopting DSP (see Methods) and adjusting the power of signal light and LO light. QPSK signal, one of the most widely used modulation formats in coherent optical communications, is performed to detect by the OCR. Figure 5b–e summarize the measured constellation diagrams of 25, 50, 90, and 100 Gbaud, corresponding to the line rates of received data as 50, 100, 180, and 200 Gbit/s. All constellation diagrams show high-quality reception for QPSK signals, of which the BERs are lower than the soft-decision forward error correction (FEC) limit of 2 × 10^−2^. And when the data rate is not exceeding 90 Gbaud, the BERs can be below the hard-decision FEC limit of 3.8 × 10^−3^. Next, we increase the data-reception rate to 240 Gbit/s by performing 16 QAM signal reception. The constellation diagrams of 40 and 60 Gbaud 16 QAM signals are shown in Fig. 5g, h, respectively. When 60 Gbaud 16 QAM signal (data rate up to 240 Gbit/s) is performed to receive, the measured BER of 2.9 × 10^−2^ is lower than the soft-decision FEC limit^44^ of 4 × 10^−2^. As shown in Fig. 5f, a back-to-back BER test curve is performed on 50 Gbaud QPSK signals, reflecting the relationship between BER and signal light power at 7-dBm LO light. Overall, the low BERs at ultrahigh-speed data receptions benefit from the large bandwidth of the graphene-on-PSW PDs and notably improved detection sensitivity by the coherent-detection scheme.

## Discussion

Overall, the results demonstrate that our proposed graphene-based OCR exhibits ultrahigh-speed and high-quality reception for advanced modulation formats that encode information on both the amplitude and phase of the light. To our knowledge, this is the first experimental demonstration of a high-speed OCR beyond 240 Gbit/s based on graphene. Generally, the OCR has been widely used in long-range optical communication systems (usually >80 km) owing to high receiving sensitivity. However, it is challenged by the cost and power consumption of the DSP chip and the complexity of the system to be adopted in the shorter range (usually use direct detection). In our view, with the reduction of the cost of the coherent detection system (such as DSP-free technology^45^) and the increase of the transmission capacity (>1.6 Tbit/s), the OCR has the potential to cover all-range optical communication systems.

Compared to the OCRs with the conventional PDs based on III–V^46^ and Si–Ge^47^ platforms, the graphene-based OCR combines the benefits of high-speed operation and CMOS-technology compatibility. Supplementary Table 2 compares the performances with the state-of-the-art OCRs, including the number of PDs, bandwidth, receiving capacity, modulation format, and shot noise caused by dark current. The overall performances of our OCR in terms of bandwidth and receiving capacity (Gbaud) are comparable to the traditional silicon–germanium^48^ or III–V-based^49^ OCRs. The larger shot noise caused by the dark current of this graphene-based OCR may reduce the sensitivity of the OCR (See Supplementary Note 4), which can be further vanished by the unbiased photothermoelectric-effect graphene-based PDs^27^. Furthermore, the responsivity of the plasmonic waveguide-integrated graphene-based PD can be further improved by other structures^20,21^, which is closer to that of the silicon–germanium PDs. We believe that the bandwidth of graphene-on-PSW PDs can be further extended to more than 150 GHz^22^ by optimizing the contact resistance between graphene and electrodes, supporting data rates of more than 200 Gbaud. When further integrated with the on-chip polarization beam splitter, the graphene-based OCR can receive signals with data rates higher than 1 Tbit/s, for example, 16 QAM signals with the dual-polarization state at 200 Gbaud. Therefore, the proved graphene-based device provides a different-material route for ultra-compact and high-performance OCRs, competitively utilizing in the datacenters and next-generation high-speed optical interconnections.

## Methods

### Device fabrication

The devices were fabricated on a commercial silicon-on-insulator wafer with a 220-nm-thick top silicon layer and a 2-μm-thick buried oxide layer. The fabrication process is depicted in Supplementary Fig. 4. Firstly, the silicon-based passive parts, including grating couplers, MMI couplers, silicon waveguides, and silicon tapered couplers, were patterned by one-step electron-beam lithography (EBL) (Vistec EBPG 5000plus ES). And they were fabricated by inductively coupled plasma etching (Plasmalab system 100 ICP180), referring to the 220-nm height of silicon waveguides. Secondly, the PSWs were patterned by a second EBL and fabricated by electron-beam evaporation (Ohmiker-50B) followed by a lift-off process. The metal is Au (90 nm)/Ni (5 nm), and the plasmonic slot width is 100 nm. Finally, a commercial express transfer single-layer graphene grown by chemical vapor deposition was wet-transferred to the chip, patterned by a third EBL, and fabricated by reactive ion etching to form the required graphene structure. The referenced one-dimensional grating couplers, PSWs in different lengths, and referenced MMI couplers were fabricated together with the proposed OCR. The images of the fabricated chip are shown in Supplementary Fig. 5. After device fabrication, the measured Raman spectra in Supplementary Fig. 6 indicate the high quality of graphene in four PDs thanks to the simple process.

### High-speed data reception for balanced and coherent detection

As shown in Figs. 4a and 5a, the input light generated by a tunable laser at 1550 nm is divided into two beams through a 1:1 optical coupler at the signal transmitting end. One beam is used as the signal light input into the commercial IQ transmitter (coherent solutions), and the other is used as the LO light. The two independent radio frequency signals (I/Q-branch) with a length of a pseudorandom bit sequence of 2^9^ − 1 generated from a 120-GSa/s arbitrary waveform generator (Keysight M8194A) are encoded on the signal light at 1550 nm by the commercial IQ transmitter. After being amplified by the erbium-doped fiber amplifiers, the signal and the LO light are input into the OCR via two incident grating couplers. Before the signal and LO light are input into the grating couplers, a tunable attenuator is used to control the optical power, and a polarization controller is used to adjust the polarization state. The optical power of the signal and LO light entering the PDs are 0 and 7 dBm, respectively. The –0.3-V-bias voltage provided by the source meter (Keithley 2400) is applied on the PD through a bias-tee (SHF BT65 B, 65 GHz) and a high-speed ground-signal probe (50 GHz). Utilizing the PD1 and PD4 of the I-branches, a balanced detection can be realized. And a coherent detection can be realized by using the PD3 and PD4 from the I- and Q-branches, respectively. The generated electrical signals from the PDs are read out with the high-speed probes, amplified by the electric amplifiers (SHF S807 C, 55 GHz), and recorded by a real-time oscilloscope (Keysight UXR0704A, 256 GSa/s). The constellation diagrams and BERs of the BPSK, QPSK, and 16 QAM signals can be obtained utilizing the off-line DSP. Limited by the type of microwave probe, the balanced detection and coherent detection are not performed at the same time when we received the QPSK and 16 QAM signals. The power consumption (~14 fJ/bit) of the OCR is equal to the bias voltage (–0.3 V) multiplied by the sum of four PDs’ currents (~2.3 mA, each PD) and divided by the bit rate, referring to the expression of 4*IV*/bit. And considering a common condition that co-packaged with the transimpedance amplifiers in a commercial OCR, it will introduce an electric power consumption of ~1–2 pJ/bit^50,51^. Meanwhile, the optical power consumption of signal light and LO light before the grating coupler is calculated as 0.95 pJ/bit when operating at 200 Gbit/s. Therefore, these graphene-based PDs with low power consumption are suitable to be applied in the OCR, with only a negligible additional power consumption of 14 fJ/bit introduced compared to that of the transimpedance amplifiers and the LO.

### Off-line digital signal processing

In the DSP, the collected samples in two branches are first resampled at two samples/symbol. Then blind constant modulus algorithm and multi-modulus algorithm channel equalizer are used for QPSK and 16 QAM to recover the signal at one sample/symbol^42^. The residual phase noise is then compensated by the decision-aided carrier phase algorithm^52^. Finally, the BER is calculated after symbol-to-bit demapping.

## Supplementary information

Supplementary information

## Data Availability

All the data supporting this study are available in the paper and Supplementary Information. Additional data related to this paper are available from the corresponding authors upon request.

## Code availability

The simulation and computational codes for this study are available from the corresponding authors on reasonable request.
