# Supplementary material for: Ultrahigh-speed graphene-based optical coherent receiver
Source: Nat Commun. 2021 Aug 20;12:5076. doi: 10.1038/s41467-021-25374-0 (PMC8379243; doi:10.1038/s41467-021-25374-0)
Supplement: Supplementary file 1 — Supplementary information [file 41467_2021_25374_MOESM1_ESM.pdf]

Supplementary Information for

Ultrahigh-speed graphene-based optical coherent receiver

Yilun Wang[1, †], Xiang Li[2, †], Zhibin Jiang[1], Lei Tong[1], Wentao Deng[1], Xiaoyan Gao[1], Xinyu Huang[1], Hailong Zhou[1], Yu Yu[1], Lei Ye[1, *], Xi Xiao[3,4, *], and Xinliang Zhang[1, *]

[1] Wuhan National Laboratory for Optoelectronics and School of Optical and Electronic Information, Huazhong University of Science and Technology, Wuhan 430074, China.

[2] Electrical Engineering Division, School of Engineering, University of Cambridge, Cambridge CB31SE, UK.

[3] State Key Laboratory of Optical Communication Technologies and Networks, China Information and Communication Technologies Group Corporation (CICT), Wuhan 430074, China.

[4] National Information Optoelectronics Innovation Center, Wuhan 430074, China.

[†] These authors contributed equally to this work

*Corresponding email: leiye@hust.edu.cn; xxiao@wri.com.cn; xlzhang@mail.hust.edu.cn

Supplementary Note 1. Design and optical simulations of the 90-degree optical hybrid and the graphene-on-plasmonic slot waveguide photodetector.

For an ideal optical coherent receiver (OCR), the 90-degree optical hybrid should meet the following transmission matrix:

$$\begin{bmatrix} \mathbf{E}_{\text{out}_1}(t) \\ \mathbf{E}_{\text{out}_2}(t) \\ \mathbf{E}_{\text{out}_3}(t) \\ \mathbf{E}_{\text{out}_4}(t) \end{bmatrix} = \frac{1}{2} \cdot \begin{bmatrix} e^{j\varphi_{11}} & e^{j\varphi_{12}} \\ e^{j\varphi_{21}} & j \cdot e^{j\varphi_{22}} \\ e^{j\varphi_{31}} & -e^{j\varphi_{32}} \\ e^{j\varphi_{41}} & -j \cdot e^{j\varphi_{42}} \end{bmatrix} \cdot \begin{bmatrix} \mathbf{E}_{\text{in}_1}(t) \\ \mathbf{E}_{\text{in}_2}(t) \end{bmatrix} \quad (1)$$

In the matrix, the phase changes are satisfied the following relationship:

$$\varphi_{11} - \varphi_{12} = \varphi_{21} - \varphi_{22} = \varphi_{31} - \varphi_{32} = \varphi_{41} - \varphi_{42} = \varphi \quad (2)$$

Here, the 90-degree optical hybrid utilizes a common 4×4 multimode interference (MMI) coupler, which works in the self-imaging principle[1] to control the phase of the output-port light. The structure diagram of the MMI coupler is shown in Supplementary Fig. 1. In this case, the optical phase changes of the signals in the 4×4 MMI coupler are given as the following equations.

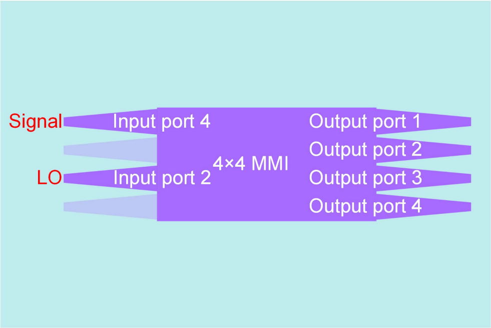

Supplementary Fig. 1 Schematic diagram of the 90-degree optical hybrid, adopting a 4×4 MMI coupler.

For $r+s$ is even,

$$\Phi_{rs} = \frac{\pi}{16}(s-r)(8+r-s)+\pi \quad (3)$$

For $r+s$ is odd,

$$\Phi_{rs} = \frac{\pi}{16}(r+s-1)(8-r-s+1) \quad (4)$$

where $r = 1, 2, 3, 4$ is the bottom-up numbering of the input ports and $s = 1, 2, 3, 4$ is the top-down numbering of the output ports, Φ_{rs} is the phase shift from the input ports to the output ports.

In the proposed OCR, the signal light is input from the input port 4, and the LO light is input from the input port 2. According to Equations (3) and (4), the phase shifts between the input ports and output ports are shown in Table S1. The corresponding transfer function of the 4×4 MMI coupler is given as follows,

$$\begin{bmatrix} \mathbf{E}_{\text{out}_1}(t) \\ \mathbf{E}_{\text{out}_2}(t) \\ \mathbf{E}_{\text{out}_3}(t) \\ \mathbf{E}_{\text{out}_4}(t) \end{bmatrix} = \frac{1}{2}\cdot\begin{bmatrix} e^{j\pi} & e^{j\frac{3\pi}{4}} \\ e^{j-\frac{\pi}{4}} & e^{j\pi} \\ e^{j\frac{3\pi}{4}} & e^{j\pi} \\ e^{j\pi} & e^{j-\frac{\pi}{4}} \end{bmatrix}\cdot\begin{bmatrix} \mathbf{E}_{\text{in}_4}(t) \\ \mathbf{E}_{\text{in}_2}(t) \end{bmatrix} = \frac{1}{2}\cdot\begin{bmatrix} e^{j\pi} & -e^{j-\frac{\pi}{4}} \\ e^{j-\frac{\pi}{4}} & j\cdot e^{j-\frac{3\pi}{2}} \\ e^{j\frac{3\pi}{4}} & -j\cdot e^{j-\frac{\pi}{2}} \\ e^{j\pi} & e^{j-\frac{\pi}{4}} \end{bmatrix}\cdot\begin{bmatrix} \mathbf{E}_{\text{in}_4}(t) \\ \mathbf{E}_{\text{in}_2}(t) \end{bmatrix} \quad (5)$$

And Equation (5) satisfies the relationship in Equation (2).

$$\varphi_{14}-\varphi_{12}=\varphi_{24}-\varphi_{22}=\varphi_{34}-\varphi_{32}=\varphi_{44}-\varphi_{42}=\frac{5\pi}{4} \quad (6)$$

The simulated power imbalance (<1.5 dB) and phase deviation (<7.5°) of the MMI coupler are shown in Supplementary Fig. 2.

Supplementary Table 1. The phase shifts between input ports and output ports of the 4×4 MMI coupler. S stands for the numbering of the output port.

	S=1	S=2	S=3	S=4
Φ_{4S}	π	-π/4	3π/4	π
Φ_{2S}	3π/4	π	π	-π/4

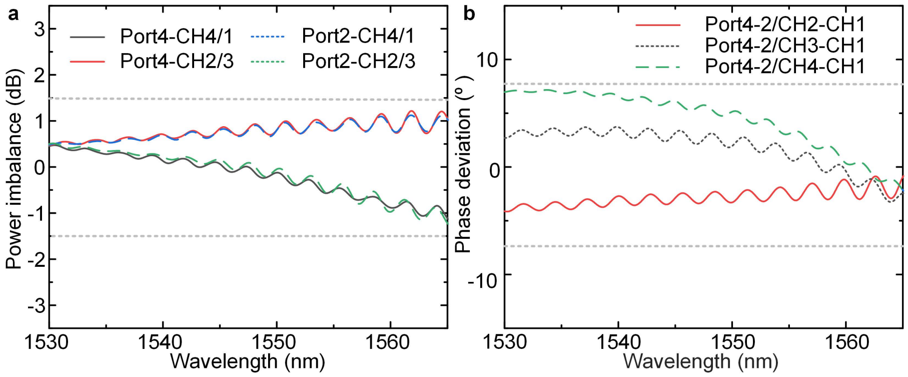

Supplementary Fig. 2 Simulated MMI coupler as a 90-degree optical hybrid. a Simulated power imbalances of the optical hybrid. Port 4/2 represents the input port, CH1/2/3/4 stands for the output port. **b** Simulated phase deviations between different output ports of the optical hybrid, as the light simultaneous input into the input port 4 and 2.

The design and optical simulations of graphene-on-plasmonic slot waveguide (PSW) photodetector (PD) are similar to the previous work[2], and a surface conductivity model is treated for graphene. Here, we mainly define the active detection area of the graphene-on-PSW PD more accurately. The in-graphene-plane field component is responsible for the absorption by graphene.[3] As shown in the Supplementary Fig. 3b, the distribution of the in-plane electric field $|\mathbf{E}_{x,y}|$ at the graphene boundary is calculated, which is the white line signed in the simulated in-plane electric field $\sqrt{|\mathbf{E}_x|^2+|\mathbf{E}_y|^2}$ distribution (Supplementary Fig. 3a). And we calculate the $|\mathbf{E}_{x,y}|^2$ which is proportional to the absorption and plot it in the Supplementary Fig. 3c. By doing the line integration in the 100-nm gap and outside the gap, we obtain the absorption portions in two areas to be 98.9% and 1.1%, respectively. Thus, we define the active detection width of graphene is equivalent to ~100 nm. As mentioned above, the active detection area of the graphene-on-PSW PD is 15 μm×100 nm.

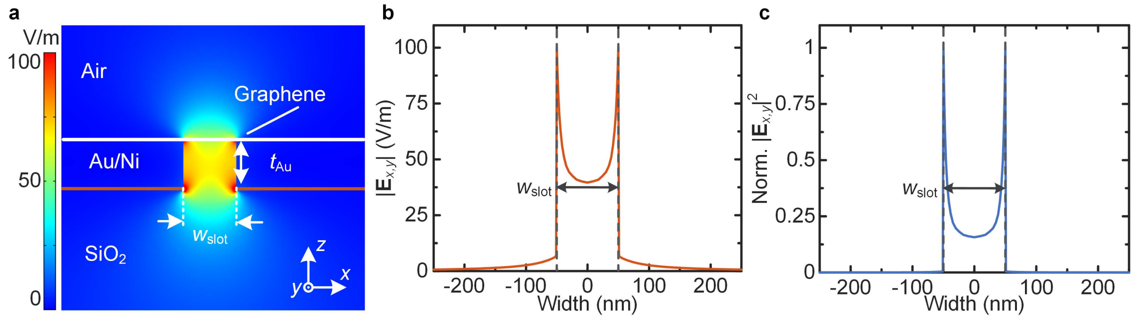

Supplementary Fig. 3 Optical simulations of graphene-on-PSW PD. a The simulated in-plane electric field $|\mathbf{E}_{x,y}|$ distribution at the cross section of the graphene-on-PSW PD. **b-c** The distribution of the electric field $|\mathbf{E}_{x,y}|$ (**b**) and the normalized $|\mathbf{E}_{x,y}|^2$ (**c**) at the graphene boundary in x direction.

Supplementary Note 2. Device fabrication and characterizations of the proposed graphene-based optical coherent receiver

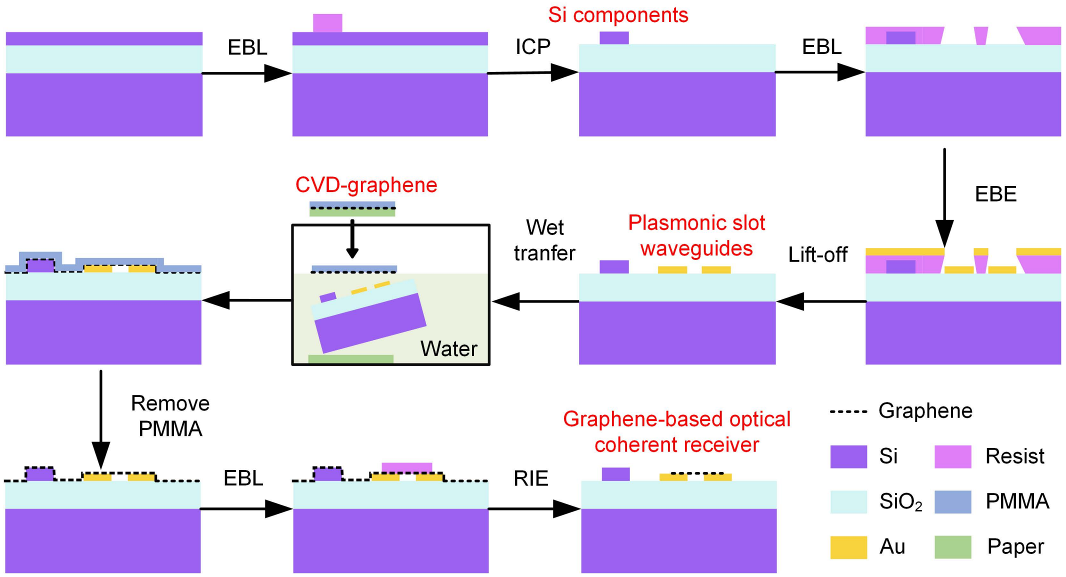

Supplementary Fig. 4 Fabrication process of the proposed OCR. EBL: electron-beam lithography, ICP: inductively coupled plasma, EBE: electron-beam evaporation, CVD: chemical vapor deposition, PMMA: polymethyl methacrylate, RIE: reactive ion etching.

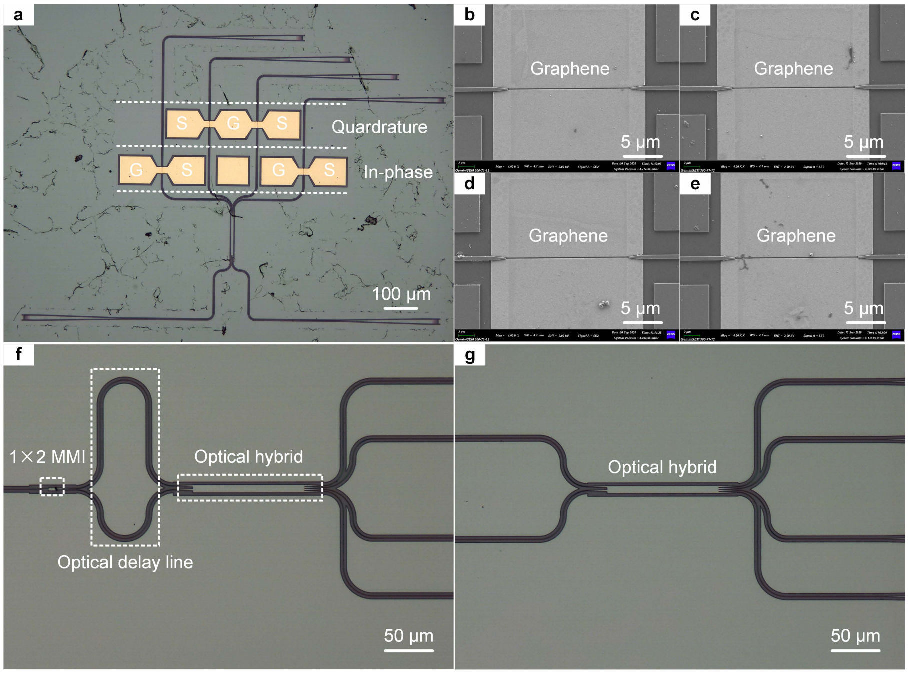

Supplementary Fig. 5 a Microscope image of the fabricated OCR. **b-e** SEM images of the four PDs after testing. **f** Microscope image of the optical hybrid cascaded with a 1×2 MMI coupler and an optical delay line. **g** Microscope image of the referenced optical hybrid.

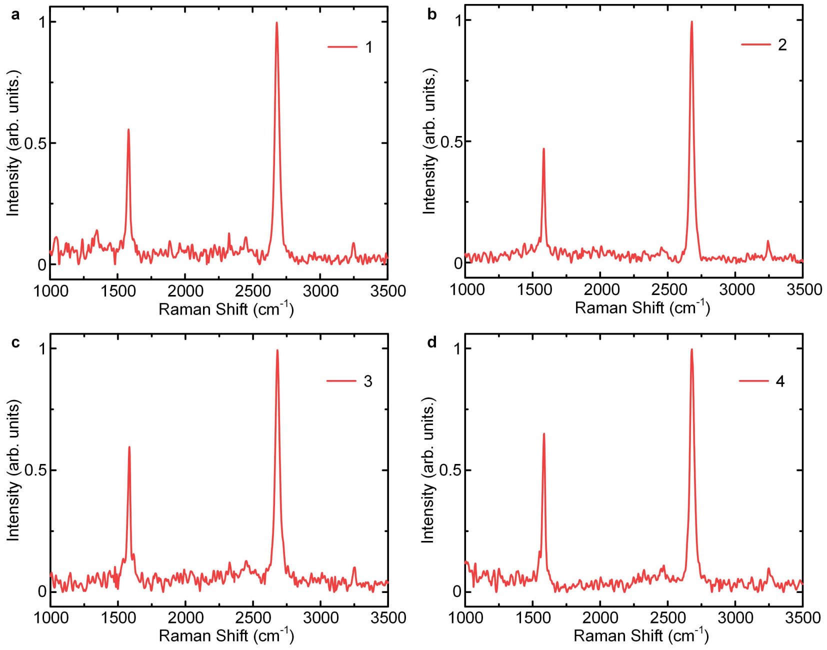

Supplementary Fig. 6 Raman spectra of the CVD-grown single-layer graphene in four PDs after the reactive ion etching process.

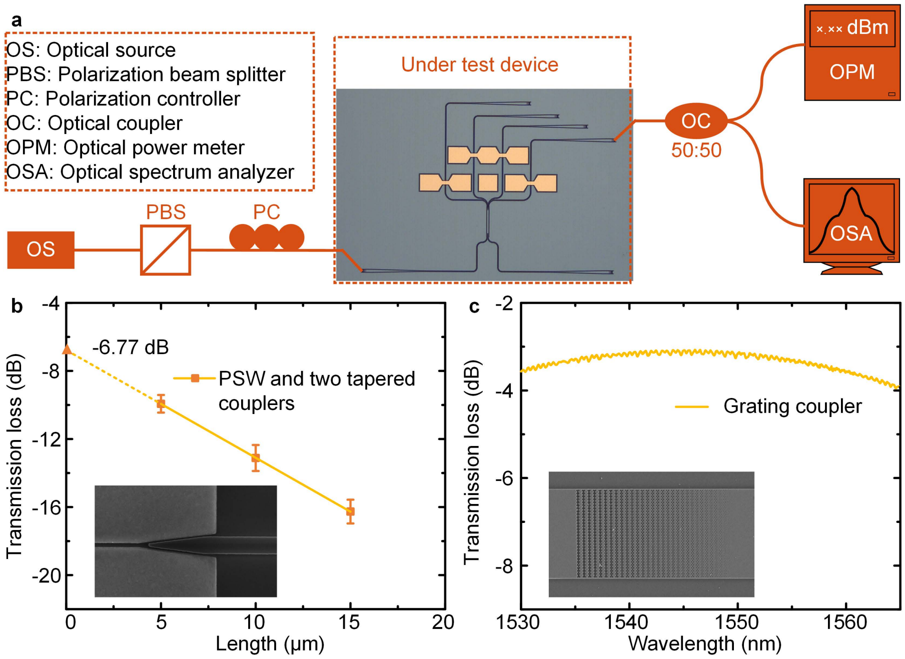

Supplementary Fig. 7 Transmission characterizations. a Experimental setup for the transmission loss measurement. **b** Transmission losses of the different-length PSWs without graphene, including two silicon-plasmonic tapered couplers. Inset is the SEM of this tapered coupler. The error bars are standard deviations of transimission loss in three lengths. **c** Transmission losses of the single referenced grating coupler. Inset is the SEM of this grating coupler.

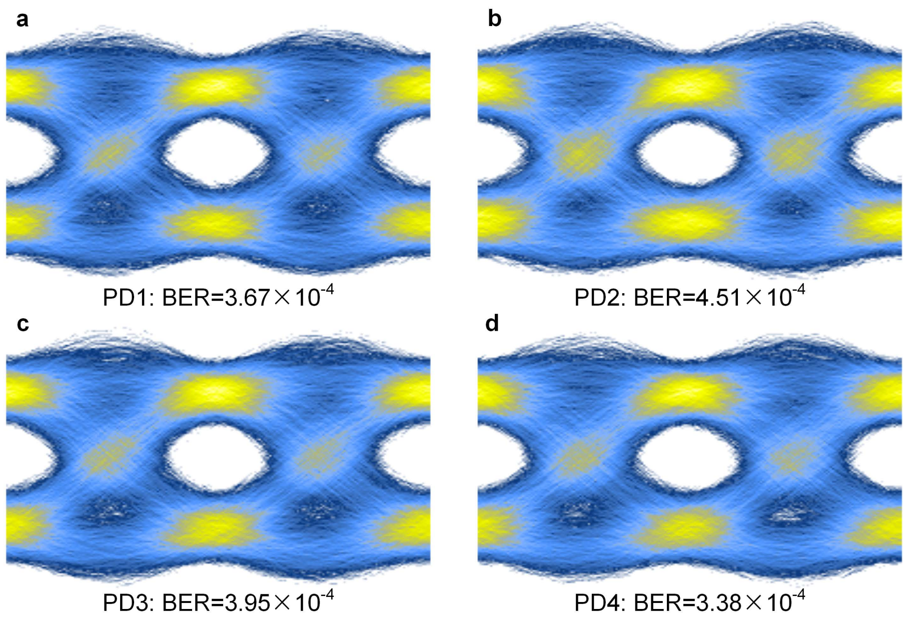

Supplementary Fig. 8 Individually reception of 72 Gbit/s non-return-to-zero signal for four PDs.

Supplementary Note 3. Photodetection contribution of the graphene-on-plasmonic slot waveguide photodetector

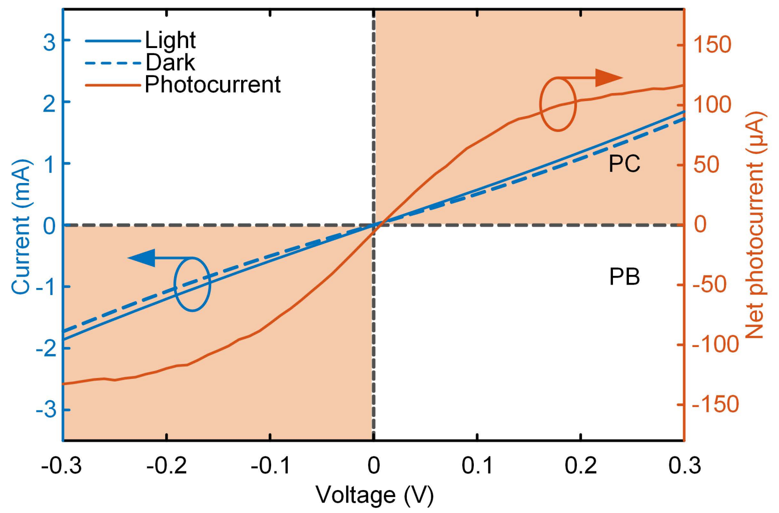

Supplementary Fig. 9 Photodetection contribution. Measured changes of current as a function of the bias voltage, including light incidence, dark incidence, and net photocurrent. PC: photoconductive, PB: photobolometric. An increase of the net photocurrent under bias indicates a dominant PC effect[4].

Supplementary Note 4. The state-of-the-art optical coherent receivers

Supplementary Table 2. A summary of the state-of-the-art OCRs. (Device 1 and 2 include packaging). PDM: polarization division multiplexing.

Device	Materials	Number of PDs	Bandwidth (GHz)	Signal reception (Gbaud)	Signal reception (Gbit/s)	Modulation format	Shot noise caused by dark current
1[5]	InP	8	50	90	800	PDM-64 QAM	~3.6 nA
2[6]	SiGe	8	52	96	768	PDM-16 QAM	~36 nA
This work	Graphene	4	67	100<br>60	200<br>240	QPSK<br>16 QAM	~14 μA

As mentioned in the reference[7], the ultimate sensitivity of the OCR is limited by the amount of shot noise, thermal noise and the residual beating of the LO with relative intensity noise. According to the concept of OCR input referred noise variance in the analytical model, shot noise cannot be addressed by the balanced detection, which acts as a constant noise floor. The thermal noise which mainly depends on the TIA and the relative intensity noise which can be addressed by the balanced detection are not discussed here.

The shot noise power spectral density for each PD is expressed as $\overline{i_{\mathrm{sn}}^2} = 2q\Delta f(\overline{i_{\mathrm{d}}} + \overline{i_{\mathrm{p}}})$, where q is the electron charge, Δf is the bandwidth of the OCR, $\overline{i_{\mathrm{d}}}$ is the dark current and $\overline{i_{\mathrm{p}}}$ is the net photocurrent. The shot noise can influence the signal-to-noise ratio (SNR) based on the expression of $\overline{i_{\mathrm{sn}}^2} / \overline{i_{\mathrm{p}}^2} = 2q\Delta f \overline{i_{\mathrm{d}}} / \overline{i_{\mathrm{p}}^2} + 2q\Delta f / \overline{i_{\mathrm{p}}}$. It is obvious that the lower dark current and the larger net photocurrent will lead to a larger SNR and a higher sensitivity of the OCR. We define the shot noise caused by the dark current ($2 \cdot \sqrt{2q\Delta f \overline{i_{\mathrm{d}}}}$) of different OCRs in Supplementary Table 2. The graphene-based PD adopted in the graphene-based OCR suffers from a large dark current (~2–3 mA) due to the biased photoconductive effect, but combines high-speed operation with a simple fabrication process and control. The shot noise caused by the dark current of this graphene-based PD may reduce the sensitivity of the OCR, and a higher input optical power is required to improve the SNR. In the future,

the dark current can be vanished by using the unbiased photothermoelectric effect-based graphene PD[8] with a little complex fabrication process. Moreover, by optimizing the optical power of the LO and signal light, we have obviously improved the SNR of the OCR, and demonstrated high-speed and high-quality receptions of advanced modulation formats in the graphene-based OCR for the first time.

Supplementary References

1. Soldano, L. B. & Pennings, E. C. M. Optical multi-mode interference devices based on self-imaging: principles and applications. *J. Lightwave Technol.* **13**, 615-627 (1995).
2. Wang, Y. *et al.* Ultra-compact high-speed polarization division multiplexing optical receiving chip enabled by graphene-on-plasmonic slot waveguide photodetectors. *Adv. Opt. Mater.* https://doi.org/10.1002/adom.202001215 (2021).
3. Ansell, D. *et al.* Hybrid graphene plasmonic waveguide modulators. *Nat. Commun.* **6**, 8846 (2015).
4. Ma, P. *et al.* Plasmonically enhanced graphene photodetector featuring 100 Gbit/s data reception, high responsivity, and compact size. *ACS Photonics* **6**, 154-161 (2018).
5. Lal, V. *et al.* 1.6Tbps coherent 2-channel transceiver using a monolithic Tx/Rx InP PIC and single SiGe ASIC. In Proc. *Optical Fiber Communication Conference* M3A.2 (Optical Society of America, San Diego, 2020).
6. Yamanaka, S. *et al.* Silicon photonics coherent optical subassembly with EO and OE bandwidths of over 50 GHz. In Proc. *Optical Fiber Communication Conference* Th4A.4 (Optical Society of America, San Diego, 2020).
7. Zhang, B., Malouin, C. & Schmidt, T. J. Design of coherent receiver optical front end for unamplified applications. *Opt. Express* **20**, 3225-3234 (2012).
8. Marconi, S. *et al.* Photo thermal effect graphene detector featuring 105 Gbit s^{-1} NRZ and 120 Gbit s^{-1} PAM4 direct detection. *Nat. Commun.* **12**, 806 (2021).
